# Mechanical Properties of Ternary Composite from Waste Leather Fibers and Waste Polyamide Fibers with Acrylonitrile-Butadiene Rubber

**DOI:** 10.3390/polym15112453

**Published:** 2023-05-25

**Authors:** Le Thuy Hang, Quoc-Viet Do, Luu Hoang, Luc The Nguyen, Nguyen Pham Duy Linh, Vu Anh Doan

**Affiliations:** 1 Faculty of Garment Technology and Fashion Design, Hung Yen University of Technology and Education, Hung Yen 160000, Vietnam; hanglt1983@gmail.com (L.T.H.); luugiaphucloc@gmail.com (L.H.); nguyentheluc82@gmail.com (L.T.N.); 2 School of Material Science, Japan Advanced Institute of Science and Technology, 1-1 Asahidai, Ishikawa, Nomi 923-1292, Japan; 3 Center for Polymer Composite and Paper, Hanoi University of Science of Technology, Hanoi 100000, Vietnam

**Keywords:** acrylonitrile-butadiene rubber, fiber reinforced composite, waste leather, waste polyamide

## Abstract

This study aimed to improve the mechanical properties of a composite material consisting of waste leather fibers (LF) and nitrile rubber (NBR) by partially replacing LF with waste polyamide fibers (PA). A ternary recycled composite NBR/LF/PA was produced by a simple mixing method and vulcanized by compression molding. The mechanical properties and dynamic mechanical properties of the composite were investigated in detail. The results showed that the mechanical properties of NBR/LF/PA increased with an increase in the PA ratio. The highest tensile strength value of NBR/LF/PA was found to have increased about 1.26 times, that is from 12.9 MPa of LF50 to 16.3 MPa of LF25PA25. Additionally, the ternary composite demonstrated high hysteresis loss, which was confirmed by dynamic mechanical analysis (DMA). The presence of PA formed a non-woven network that significantly enhanced the abrasion resistance of the composite compared to NBR/LF. The failure mechanism was also analyzed through the observation of the failure surface using scanning electron microscopy (SEM). These findings suggest that the utilization of both waste fiber products together is a sustainable approach to reducing fibrous waste while improving the qualities of recycled rubber composites.

## 1. Introduction

The textile industry is one of the largest and most important industries worldwide, producing a vast array of products ranging from clothing and household textiles to industrial materials [1]. However, this industry also generates a significant amount of fibrous waste that is non-decomposable and perpetually contaminates the soil and groundwater system [2,3]. One such material is polyamide (PA) fibers, which are widely used in textile products due to their durability, strength, and versatility. Moreover, the substantial cumulating PA waste products makes them a serious environmental concern [4]. The estimated annual waste volume of PA in the world is approximately 200,000 tons [5]. Due to its substantial rise in demand, exceeding that of other plastic materials in recent years, it is important to incorporate this product category into sustainable development strategies. As a result, researchers developed various technologies to recycle this material, such as chemical recycling and mechanical recycling (remelting).

Chemical recycling aims to recover and reuse monomers as its primary objective. In this method, the reaction of PA with decomposing agent was determined by the existence of polar amide groups in the main polyamide chain. The depolymerization reaction could be ammonolysis [6], hydrolysis [7], and glycolysis [8]. The objective of PA depolymerization is to produce hexamethylenediamine and caprolactam, both of which can be effectively employed in the synthesis of new polyamides or other polymers. Mechanical recycling is considered the most straightforward method for recycling PA. Among the techniques with potential application, melt extrusion stands out as a viable approach. However, since polyamides are thermoplastic polymers, they cannot be further used after a limited number of times, as their properties are not maintained. Therefore, having well-defined and repetitious properties for recycled products, even after undergoing multiple processes, is highly significant. Lozano-González et al. [9] investigated the effect of recycling cycles on the physical and mechanical properties of nylon 6 using injection molding. The study demonstrated that PA6 can undergo up to seven cycles of injection molding without experiencing any notable deterioration in its physical and mechanical properties. Another sustainable approach to recycling is the utilization of waste fibers for the fabrication of composite materials, which proves beneficial from both an environmental and material development perspective [10,11,12,13].

Fundamentally, the incorporation of fiber reinforcement and polymer matrix results in composite materials that exhibit remarkable stiffness, strength, and toughness. The reinforcing fibers are typically categorized into three groups: “natural fibers” (i.e., bamboo, kenaf, sisal, jute) [14,15], “synthetic fibers” (i.e., carbon fibers, glass fibers, aramid fibers) [16] and “animal-based fibers” (i.e., wool, silk, leather fiber) [17]. The polymer matrix plays a crucial role in connecting the fibrous reinforcement from the surroundings and facilitating the appropriate alignment of the fibers. G.Jayalatha et al. [18] fabricated composite polystyrene(PS)/natural rubber(NR) with nylon-6 fibers by melt mixing method. The fibers were also treated by resorcinol formaldehyde latex (RFL) to enhance adhesion with matrix. The results showed that improving interphase adhesion between the fibers and PS/NR ensured the transfer of stress from the comparatively less sturdy matrix to the fiber and, thus, enhanced the mechanical properties. Composite of natural rubber (NR) and short nylon fiber were also investigated by S. Kutty et al. [19,20] and Senapati et al. [21]. These studies indicated that the incorporation of short nylon fibers into the composite significantly enhances its mechanical properties, specifically in terms of tensile strength and tear strength. However, a disadvantage is that the non-polar nature of NR results in weak interaction with the fibers, as reported in previous research [22]. To overcome this limitation, other polar rubbers, such as styrene-butadiene rubber (SBR) [23] and acrylonitrile-butadiene rubber (NBR) [24], were used as base polymers in fiber-reinforced composites due to their favorable adhesion with nylon fibers. On the other hand, C. Rajesh’s group also investigated thermal and dielectric properties of nylon-6-reinforced NBR [25,26]. These studies showed that the improvements were not limited to mechanical properties, but also extended to thermal and dielectric properties of the composite.

In our previous work [27], a significant amount of waste leather fiber (LF) was introduced into NBR matrix to prepare recycled composite. The findings from that study identified the optimal LF/NBR ratio for reinforcement as 50/50. Building upon these results, the present study maintains this ratio and partially replaces it with waste PA fibers. As well as the recycling purpose, the objective of incorporating waste PA short fibers into LF/NBR compound is to enhance mechanical properties of LF/NBR composite. The curing behavior, tensile strength, and abrasion resistance, as well as the dynamic mechanical properties, were also thoroughly investigated and compared to those of the LF/NBR composite and pure NBR.

## 2. Materials and Methods

### 2.1. Material

Acrylonitrile–butadiene rubber (NBR), with acrylonitrile content of about 33%, was purchased from Kumho (Seoul, Korea). The waste leather was collected from Vietnamese Leather factories (Hung Yen industrial area). The waste polyamide fibers (PA) were collected from textile factories in Vietnam. Zinc oxide (ZnO) and stearic acid are commercial chemicals that were supplied from Henan Kingway Chemical Co., Ltd. (Zheng Zhou, China). Sulfur (S) and accelerator (TBBS) were bought from Lanxess (Köln, Germany).

### 2.2. Preparation of Ternary Composite NBR/LF/PA

The leather was first reduced in size following the procedure in our previous work [27] to obtain waste leather fiber (LF). The grinding process was conducted by a hammer mill operating at a rotor speed of 2000 rpm. The mill consisted of six rows of hammers and using a 40-mesh screen. The obtained waste PA fibers were then cut to an average of 40 mm in length. Both of waste fibers were dried by oven at 80 °C for two hours before the mixing process. The structure of LF and PA were characterized by FT-IR method and the results are shown Figures S1 and S2 (Supplementary Materials). The ternary composite was prepared by mixing NBR/LF/PA with curatives as shown in Table 1.

The mixing was conducted using an internal mixer (Toyoseiky Labo Plastomil; Tokyo, Japan). Initially, NBR was masticated at 125 °C for 2 min to reduce its viscosity. Then, waste LF and PA were added gradually, in the ratios outlined in Table 1, to achieve a uniform dispersion. The curatives were added last to the compound, this process continued for 3 min. The rotor speed was controlled at 50 rpm. After completing the mixing process, flat sheets of the compound were obtained by using a two-roll mill with a set nip gap of 1.5 mm. The samples were vulcanized at 150 °C and 10 MPa by a compression molding for 20 min.

### 2.3. Measurements

Curing behaviors were analyzed by a Moving Die Rheometer (Toyoseiky RLR-4; Tokyo Japan) at 150 °C according to ISO 6502. The tensile and tear measurement were conducted by using a tensile testing machine (Intron 5582; Norwood, MA, USA). The testing procedure for tensile and tear was following the ASTM D412-D and ASTM D624-C standards, respectively. The abrasion test was performed by a rotary drum abrasion tester (GOTECH GT-7012-DA; Taichung, Taiwan) according to the DIN-53516. The normal force was controlled at 5N by a constant weight. The rotational speed of the cylinder was fixed at 40 rpm to ensure a rubbing length of 40m. Sandpaper with 60 grit was used in this abrasion test. After the wearing process, the samples were cleaned using a soft brush to remove the wear debris. The abrasion resistance was calculated from the difference in weight before and after the test. Three measurements were conducted for each sample. Morphology of fracture surfaces after tensile and abrasion test were observed by scanning electron microscopy (Jeol JSM-6360LV; Tokyo, Japan). The samples were coated with platinum using a coating machine (Jeol JEC-3000 FC; Tokyo, Japan). The accelerating voltage was 20 kV. Dynamic mechanical properties were evaluated by a dynamic mechanical analyzer (TA Instrument DMA-800; New Castle, DE, USA). The samples were measured in tensile mode at frequency of 1Hz. The ramp temperature was from −90 °C to 30 °C and the heating rate was 2 °C/min.

## 3. Results and Discussion

### 3.1. Vulcanization Chracteristic

**Figure 1:**
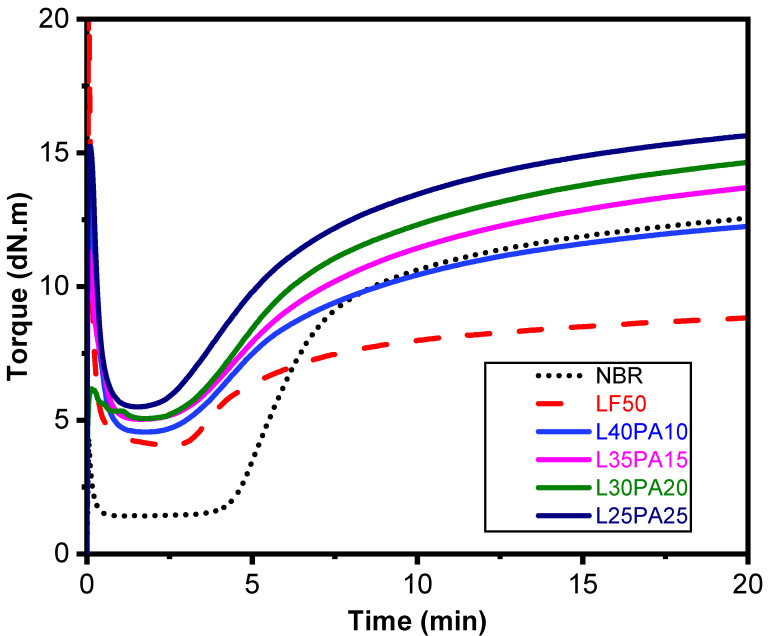
Curing curves at 150 °C.

From the curing curves shown in Figure 1 and the parameters in Table 2, both of values minimum torque (M_L_) and maximum torque (M_H_) increased with the increase in PA content in the NBR/LF compound. It was found that the scorch time (ts_2_) decreased when LF and PA were introduced. This could be ascribed to the presence of reactive functional groups in both type of fibers that act as activators and accelerate the rate of the vulcanization, as pointed out in our previous work [27]. This observation was also similar to the report of T.D. Sreeja et al. [20] and Ismail et al. [28], where an increase in filler content resulted in a decrease in scorch time. Additionally, we found that the introduction of LF to NBR led to a decrease in the value of M_H_ as the LF ratio increased. However, in the present study, we observed a different trend, where a higher PA content resulted in a higher M_H_ value. This finding is noteworthy because it suggests that the presence of PA fibers could strongly enhance the total of crosslinking in the composite. Furthermore, the value of ΔM was also higher, indicating a higher crosslink density. The enhancement of crosslink density could be proved through the good interphase adhesion between NBR and PA (Supplementary Materials Figure S4).

### 3.2. Mechanical Properties of Composite

**Figure 2:**
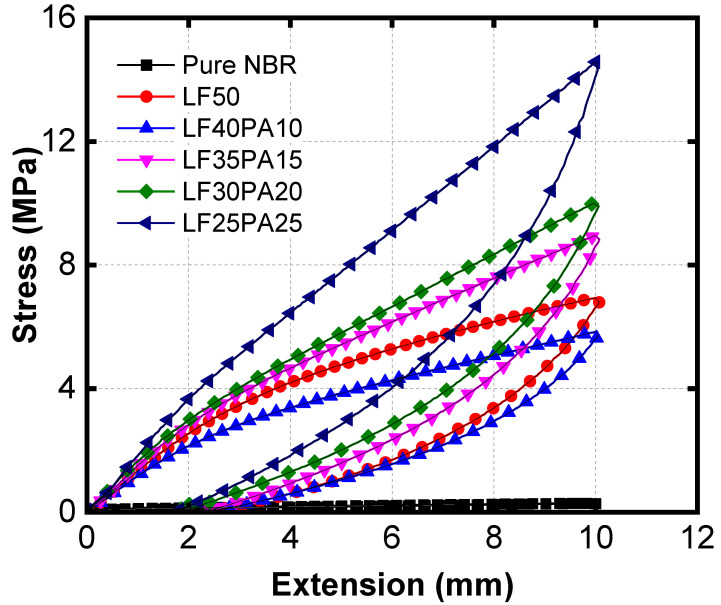
Hysteresis curve of NBR and samples with various LF/PA ratios.

**Figure 3:**
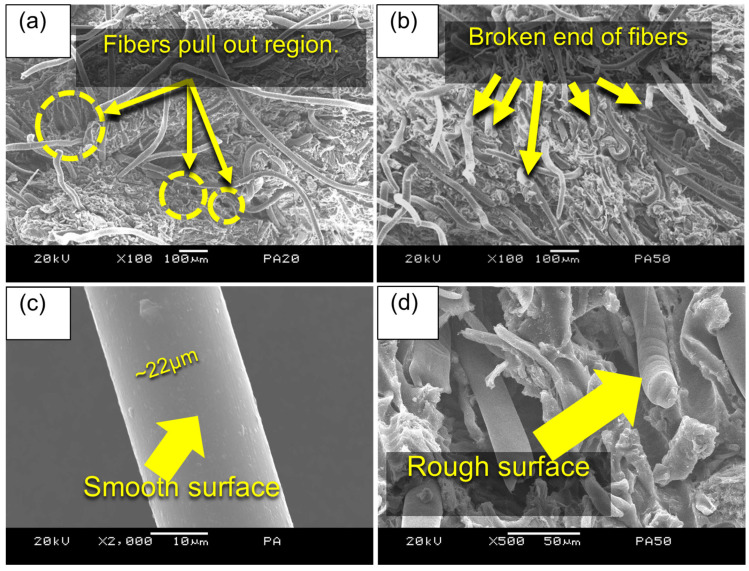
SEM image of tensile fractured surface (**a**) LF40PA10 and (**b**) LF25PA25 at ×100 magnification. (**c**) Original surface of waste PA fiber; (**d**) fractured surface of LF25PA25 at ×500 magnification.

According to previous studies [27], LFs demonstrated efficient reinforcement due to their good interaction with NBR. In our studies, the ground LFs exhibited a fiber bundle structure with weak collagen fibrils that can be easily untwisted (Supplementary Materials Figure S3). As the collagen fibrils untwist, voids easily form, leading to failure through crack propagation [29]. This represents the breaking phenomenon of LF-reinforced NBR composite. In our current research, PAs were introduced as long fibers to overcome the drawback of LF reinforcement. As shown in Table 3, the tensile strength gradually increased when waste PA fiber was introduced. The previous studies showed that the rubber reinforced with short fibers exhibited the remark mechanical anisotropy [30]. S. Soltani et al. [31] reported that the incorporation of virgin nylon fiber into NBR brought greater tensile strength than waste nylon fiber because of the high aspect ratio (L/D). In this study, the average length (L) of waste PA fiber was ~40mm, much higher than the diameter (D) value, which was around 22 μm (Figure 3c). Therefore, the long PA fibers were distributed as tangles after mixing. These tangled fibers included links, knots, and braids that acted as a non-woven fabric network. When the sample was stretched, the load was not only dissipated by friction through the tangle movement but was also transferred through the non-woven network. Thus, the reinforcing efficiency was improved. The values of elongation at break of NBR/LF/PA with various PA ratios fluctuated around 50%. It was much lower than elongation at break of pure NBR but still higher than the value of LF50. It is well known that an increased amount of filler causes a decrease in deformation by restricting the mobility of chains [32,33]. In this case, elongation at break of samples having PA fibers was higher than sample LF50 because of the slippage phenomenon occurring between fibers and matrix. A high L/D ratio results in PA fibers slipping before failure. These results are also consistent with the findings in other works [34,35,36]. The tear strength of the ternary composite was found to be significantly improved when mixed with waste PA fibers. Similar to the tensile strength, an increase in the amount of PA fibers led to an increase in the tear strength value at all ratios. This is because tear strength is related to crack propagation, and the presence of PA coils tends to hinder the growth of micro-cracks, thereby increasing the tear strength of the composite material. [34,37]. Furthermore, the incorporation of any filler into the matrix induces the energy dissipation which was reported [38,39]. The friction caused by the movement of PA fibers in polymer matrix will generate heat and increase the hysteresis loss [21]. The hysteresis curves in Figure 2 show the loss energy during deformation, the area under curve corresponds to the energy dissipation. Obviously, the value of area under curve in Table 3 shows an increase in hysteresis loss when there was an increase in the PA fibers’ proportion.

Figure 3 shows the SEM image of the fracture surface of composite and the surface of PA fibers before mixing. As illustrated in Figure 3a,b, the fracture surfaces of LF40PA40 and LF25PA25 are presented, respectively. The observation indicates that the sample with a lower PA content (Figure 3a) displays several holes, which serves as evidence of the presence of slippage between fibers and the polymer matrix. In the LF25PA25 sample, there were many broken ends of fiber chains instead of holes (Figure 3b). This was attributed to the limitation of movement space. High PA content reduces the possibility of movement of the material under applied stress. Interestingly, the results also showed that the PA fibers were longitudinally oriented with stress direction which was also found in other research [40]. This is reasonable based on the above explanation about the high value of L/D. Figure 3d also illustrates the very good load-carrying ability of the PA fibers. The surface of waste PA fibers before mixing in Figure 3c was quite smooth, while it changed to rough after the tensile test (Figure 3d). This suggests that the non-woven networking formed by PA fibers acted as an efficient load carrier. Therefore, more energy was transferred to deformed PA fibers before failure [41].

**Figure 4:**
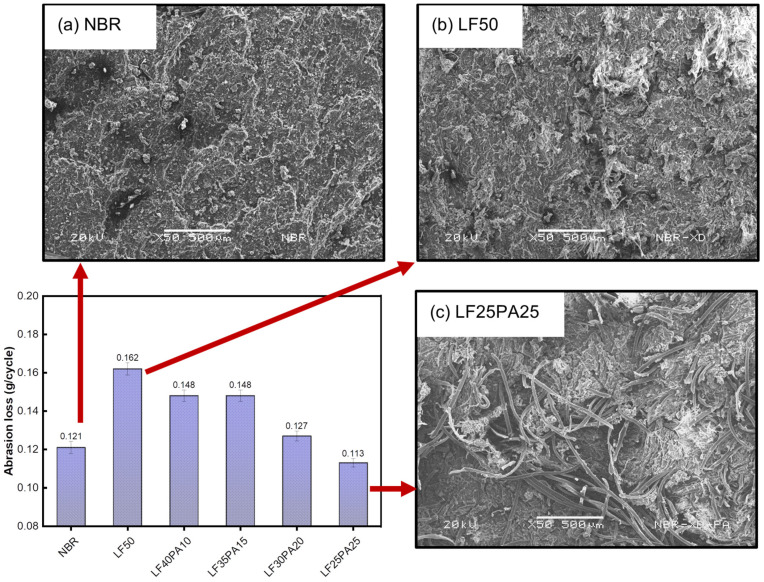
Abrasion loss of samples with various fiber ratios and worn surface of (**a**) NBR, (**b**) LF50, and (**c**) LF25PA25.

Figure 4 displays the mass loss of the samples from the abrasive experiment. The mass loss of the NBR/LF composite increased sharply compared to pure NBR. Figure 4a,b show the worn surfaces of NBR and LF50, respectively. Sample LF50 exhibited numerous curled debris, whereas NBR displayed distinctive “waves of detachment”, as evident from the observations. Previous research showed that the wear mechanism of rubber involves the breakdown of molecular structure and the rupture of local mechanics [36,37]. In this case, the significant increase in mass loss of the LF50 sample could be explained by the weak cohesion of the leather fiber-bundles under frictional force. When PA fibers were present in the samples, the weight loss decreased with increasing PA content. This can be attributed to the layer of PA in the composite, which creates networks with higher durability, slowing down the abrasion process.

### 3.3. Dynamic Mechanical Properties

**Figure 5:**
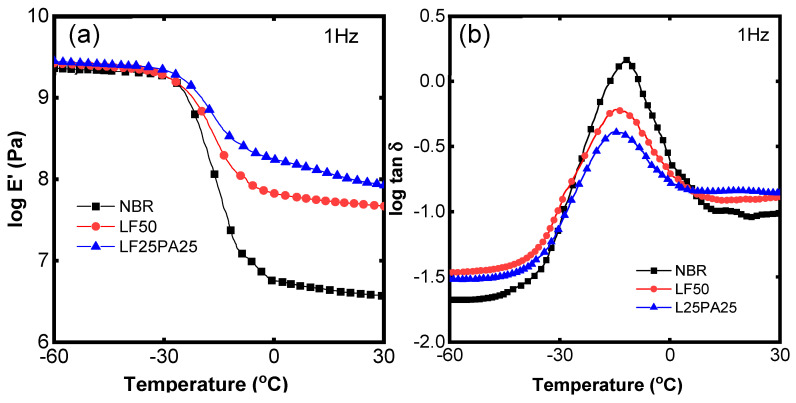
Temperature dependence of tensile storage modulus (**a**) and tan δ (**b**).

Figure 5 illustrates the dynamic mechanical properties of the samples. The storage modulus in the glassy region was slightly higher compared to that of NBR, LF50, and LF25PA25. However, in the rubbery region, the storage modulus was significantly improved in LF50 and LF25PA25 (Figure 5a). The storage modulus G’ in the rubbery region of LF25PA25 was found to have increased by 1.23 times compared to LF50, which was reasonable with data of the tensile strength. In addition, the tan δ values of the samples in Figure 5b decreased in the presence of LF and PA. The decrease in the tan δ peak occurred due to the fibers’ ability to restrict the mobility of polymeric chains. These results are consistent with another study [38]. Furthermore, the value of tan δ in the rubbery region also indicates that NBR/LF/PA exhibited higher energy dissipation compared to NBR and NBR/LF.

## 4. Conclusions

In this study, the ternary composite NBR/LF/PA was prepared using a simple mixing method with the partial replacement of waste leather fibers (LF) by waste polyamide (PA) fibers. The mechanical properties of NBR/LF/PA, such as tensile strength and tear strength, were found to be significantly enhanced in the presence of PA fibers. In particular, the tensile strength of LF25PA25 was found to have increased by about 1.26 times compared to LF50. Similarly, the tear strength was remarkably enhanced, with LF25PA25 exhibiting a value of 84.64 N/mm compared to 72.47 N/mm for LF50. Hysteresis loss data illustrated that the presence of PA also increased the energy loss of the ternary composite, which plays a crucial role in the reinforcement of PA. Dynamic mechanical properties were also examined to confirm the mechanical properties. The breaking phenomena of the composites were discussed through morphology observation to demonstrate the different reinforcing behavior of LF and PA. Moreover, the abrasion resistance of the ternary composite was significantly improved compared to the binary composite NBR/LF. This suggests that the utilization of both waste fiber products presents a truly sustainable solution for enhancing the properties of recycled materials based on nitrile rubber while also offering the benefits of recycling and cost reduction.

## Figures and Tables

**Table 1:** Formulation of NBR/LF/PA composite with various fiber ratios (in part per hundred rubber of total rubber content).

	Pure NBR	LF50	LF40PA10	LF35PA15	LF30PA20	LF25PA25
NBR	100	50	50	50	50	50
Waste leather	-	50	40	35	30	25
Waste PA	-	-	10	15	20	25
Zinc Oxide	3	3	3	3	3	3
Stearic acid	1	1	1	1	1	1
Sulfur	2.25	2.25	2.25	2.25	2.25	2.25
TBBS *	0.7	0.7	0.7	0.7	0.7	0.7

* *n-tert-butyl-2-benzothiazole sulfonamide*.

**Table 2:** Vulcanizing parameter from curing curves.

	Pure NBR	LF50	LF40PA10	LF35PA15	LF30PA20	LF25PA25
M_L_ (dN·m)	1.45	4.09	4.56	5.04	5.07	5.49
M_H_ (dN.·m)	12.55	8.81	12.24	13.69	14.63	15.63
ts_2_ (min)	4.76	3.43	2.96	2.81	2.99	2.75
tc_90_ (min)	9.82	7.25	10.52	11.23	11.75	12.88
ΔM (M_H_−M_L_)	11.10	4.72	7.68	8.65	9.56	10.14

**Table 3:** Mechanical properties of samples.

	Tensile Strength (Mpa)	Elongation at Break (%)	Tear Strength (N/mm)	Area under Curve (Area Unit)
Pure NBR	2.18 ± 0.52	357.20 ± 0.72	22.70 ± 1.30	0.22
LF50	12.90 ± 0.74	28.60 ± 0.55	72.47 ± 1.12	26.35
LF40PA10	12.01 ± 0.64	49.39 ± 0.62	73.24 ± 0.97	23.54
LF35PA15	13.62 ± 0.31	45.43 ± 0.66	75.40 ± 0.58	28.51
LF30PA20	14.92 ± 0.44	48.20 ± 0.81	83.14 ± 0.99	28.49
LF25PA25	16.30 ± 0.36	48.99 ± 0.87	84.64 ± 1.10	37.34

## Data Availability

Not available.

## Supplementary Materials

The following supporting information can be downloaded at: https://www.mdpi.com/article/10.3390/polym15112453/s1, Figure S1: FT-IR spectrum of ground waste leather (LF); Figure S2: FT-IR spectrum of waste polyamide (PA); Figure S3: Morphology of ground waste leather (LF); Figure S4: High-resolution FE-SEM images of composite NBR/LF/PA.
