# Supplementary material for: Mechanical Properties of Ternary Composite from Waste Leather Fibers and Waste Polyamide Fibers with Acrylonitrile-Butadiene Rubber
Source: Polymers (Basel). 2023 May 25;15(11):2453. doi: 10.3390/polym15112453 (PMC10255798; doi:10.3390/polym15112453)
Supplement: Supplementary file 1 [file polymers-15-02453-s001.zip › polymers-2387048-supplementary.pdf]

Supporting Information

Mechanical Properties of Ternary Composite from Waste Leather Fibers and Waste Polyamide Fibers with Acrylonitrile-Butadiene Rubber

Le Thuy Hang [1], Quoc-Viet Do [2,*], Luu Hoang [1], Luc The Nguyen [1], Nguyen Pham Duy Linh [3,*] and Vu Anh Doan [3,*]

[1] Faculty of Garment Technology and Fashion Design, Hung Yen University of Technology and Education, Hung Yen 160000, Vietnam; hanglt1983@gmail.com (L.T.H.); luugiaphucloc@gmail.com (L.H.); nguyentheluc82@gmail.com (L.T.N.)

[2] School of Material Science, Japan Advanced Institute of Science and Technology, 1-1 Asahidai, Ishikawa, Nomi 923-1292, Japan

[3] Center for Polymer Composite and Paper, Hanoi University of Science of Technology, Hanoi 100000, Vietnam

* Correspondence: doquocviet@jaist.ac.jp (Q.-V.D.); linh.nguyenphamduy@hust.edu.vn (N.P.D.L.); vu.doananh@hust.edu.vn (V.A.D.)

Keywords: acrylonitrile-butadiene rubber; fiber reinforced composite; waste leather; waste polyamide

Supporting Information

Herein, further information is provided about the FT-IR spectrum of raw polyamide fiber (PA) and ground waste leather (LF) used in this work (**Figure S1** and **Figure S2**). The morphology of LF is shown in **Figure S3**. The high-resolution FE-SEM images of NBR/LF/PA are shown in **Figure S4** to prove the good adhesion between PA fiber and NBR.

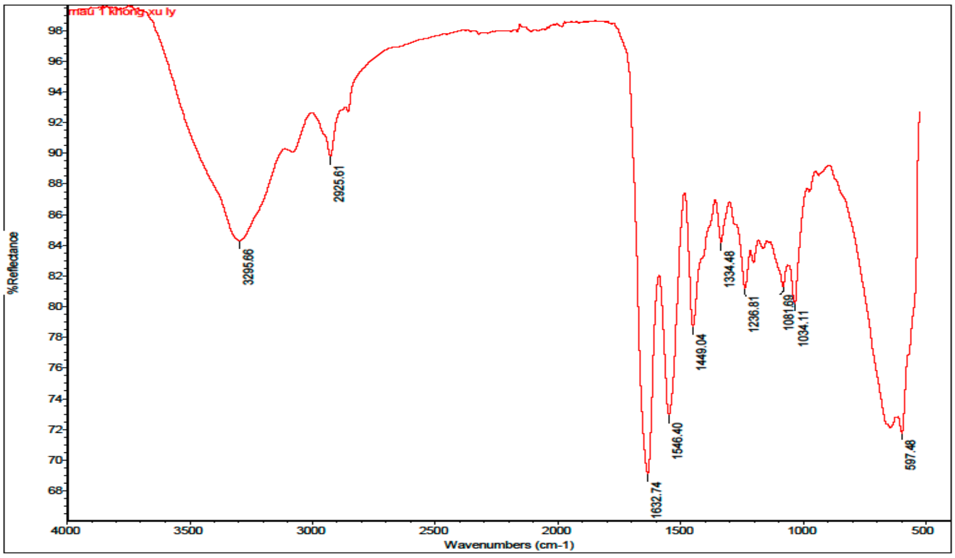

Figure S1. FT-IR spectrum of ground waste leather (LF).

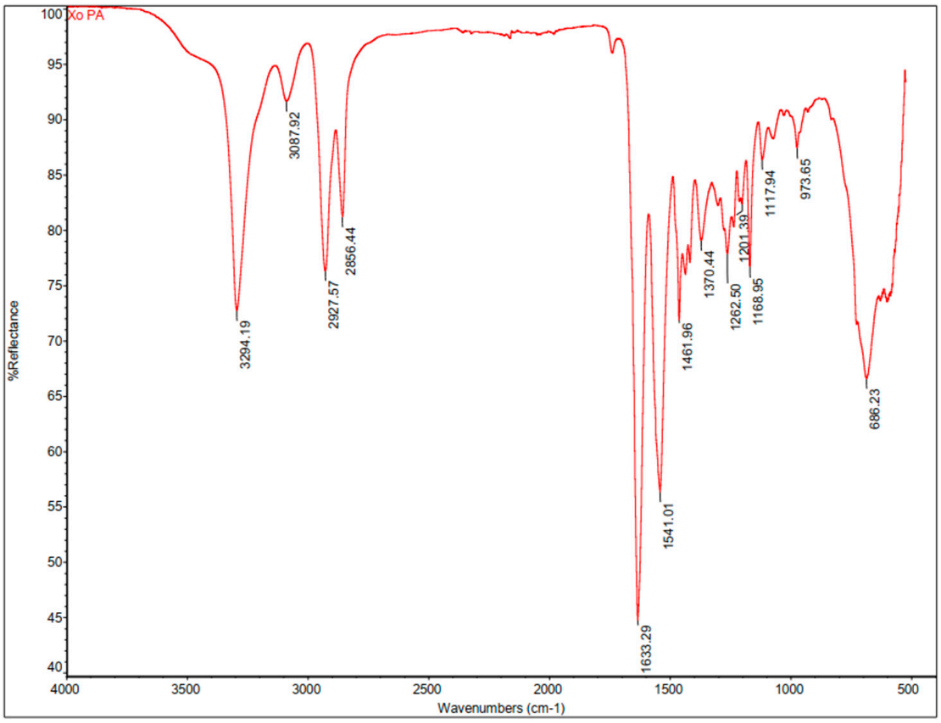

Figure S2. FT-IR spectrum of waste polyamide (PA).

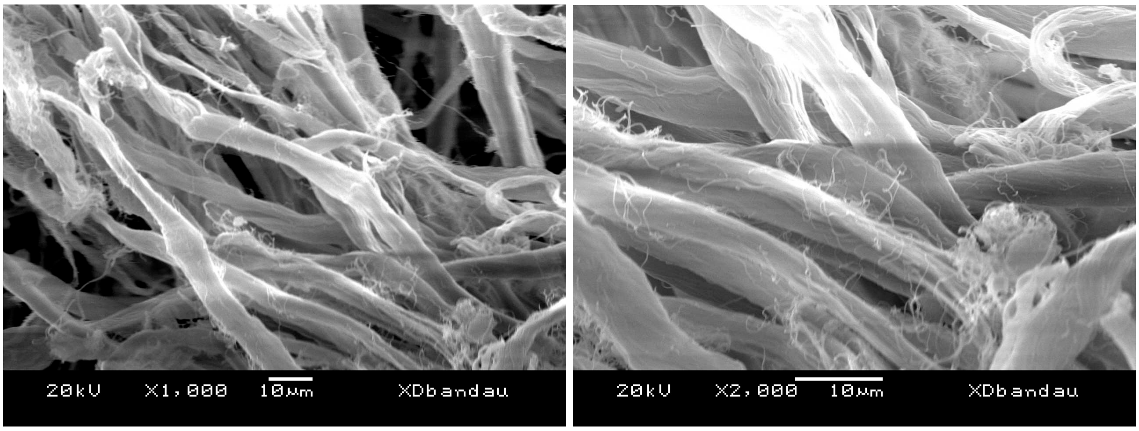

Figure S3. Morphology of ground waste leather (LF).

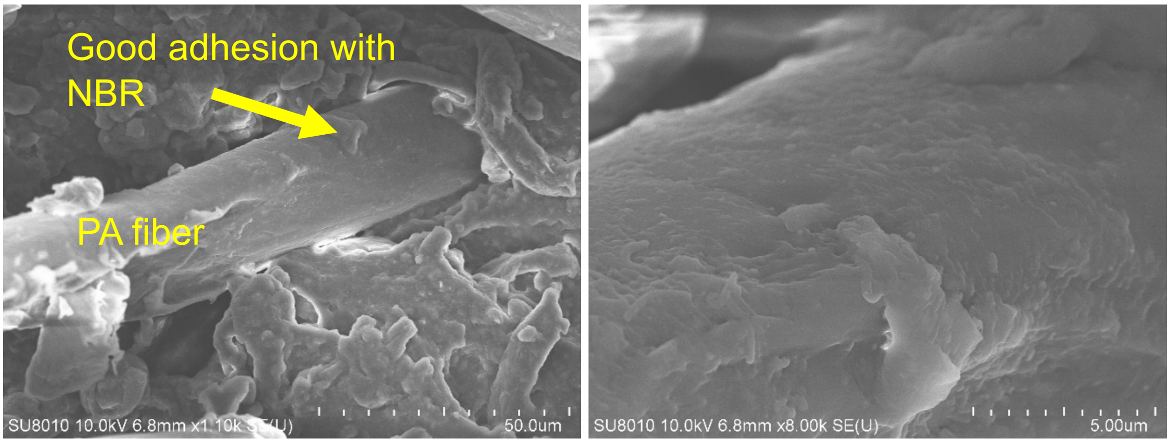

Figure S4. High-resolution FE-SEM images of composite NBR/LF/PA.
